# Simultaneous Imaging of CBF Change and BOLD with Saturation-Recovery-T_1_ Method

**DOI:** 10.1371/journal.pone.0122563

**Published:** 2015-04-23

**Authors:** Xiao Wang, Xiao-Hong Zhu, Yi Zhang, Wei Chen

**Affiliations:** Center for Magnetic Resonance Research, Department of Radiology, University of Minnesota Medical School, Minneapolis, Minnesota, United States of America; University of Pennsylvania, UNITED STATES

## Abstract

A neuroimaging technique based on the saturation-recovery (SR)-T_1_ MRI method was applied for simultaneously imaging blood oxygenation level dependence (BOLD) contrast and cerebral blood flow change (ΔCBF), which is determined by CBF-sensitive T_1_ relaxation rate change (ΔR_1_^CBF^). This technique was validated by quantitatively examining the relationships among ΔR_1_^CBF^, ΔCBF, BOLD and relative CBF change (rCBF), which was simultaneously measured by laser Doppler flowmetry under global ischemia and hypercapnia conditions, respectively, in the rat brain. It was found that during ischemia, BOLD decreased 23.1±2.8% in the cortical area; ΔR_1_^CBF^ decreased 0.020±0.004s^-1^ corresponding to a ΔCBF decrease of 1.07±0.24 ml/g/min and 89.5±1.8% CBF reduction (n=5), resulting in a baseline CBF value (=1.18 ml/g/min) consistent with the literature reports. The CBF change quantification based on temperature corrected ΔR_1_^CBF^ had a better accuracy than apparent R_1_ change (ΔR_1_^app^); nevertheless, ΔR_1_^app^ without temperature correction still provides a good approximation for quantifying CBF change since perfusion dominates the evolution of the longitudinal relaxation rate (R_1_^app^). In contrast to the excellent consistency between ΔCBF and rCBF measured during and after ischemia, the BOLD change during the post-ischemia period was temporally disassociated with ΔCBF, indicating distinct CBF and BOLD responses. Similar results were also observed for the hypercapnia study. The overall results demonstrate that the SR-T_1_ MRI method is effective for noninvasive and quantitative imaging of both ΔCBF and BOLD associated with physiological and/or pathological changes.

## Introduction

Cerebral blood perfusion through the capillary bed is essential for brain function. Imaging of cerebral blood flow (CBF) provides valuable information regarding brain physiology, function, activation and tissue viability associated with a large number of brain diseases. Arterial spin labeling (ASL), a noninvasive MRI technique which utilizes the radiofrequency (RF) pulse to label the flowing arterial water spin as an endogenous and diffusible tracer for imaging CBF [1–7], plays an ever-growing role in scientific and clinical research. An inversion-recovery preparation is commonly applied for most ASL methods, and paired images (one control and another with spin tagging) are acquired with an appropriate inversion recovery time. The signal difference between the paired images can be used to determine the CBF value and it is particularly useful for imaging relative CBF changes, for instance, induced during brain activation [8, 9] and/or physiology/pathology perturbations.

An alternative MRI-based CBF imaging method is to measure the parametric parameter of apparent longitudinal relaxation time (T_1_^app^) directly by using inversion-recovery preparation with varied inversion-recovery time (T_IR_) [2, 6, 10]. Due to the slow T_1_^app^ relaxation processing, this method requires a relatively long repetition time (TR) to acquire a serial of images with different T_IR_ values in order to generate T_1_^app^ images, thus, results in low temporal resolution for imaging CBF. Nevertheless, this method should be robust in quantifying CBF and its change in the absolute scale with the unit of ml blood/g brain tissue/min (or ml/g/min) owing to a simple relationship between T_1_^app^ and CBF (see more details in Method and Theory).

One common, interesting observation related to T_1_^app^ changes reported in the literature is the detection of T_1_^app^ increase at an early stage of ischemia, indicating a possible link between the brain tissue T_1_^app^ change and the perfusion deficit caused by the ischemia [11–14]. However, the quantitative relationship between the observed T_1_^app^ change and the CBF reduction extent caused by acute ischemia has not been rigorously, quantitatively studied. A potential difficulty for quantifying CBF via the measurement of T_1_^app^ lies on the confounding effect from the brain tissue temperature change owing to physiological and/or pathological perturbation, which can also contribute to the T_1_^app^ change [15–18]. Consideration and correction of this confound effect might improve the accuracy of quantifying CBF based on the T_1_^app^ measurement. Another layer of complexity is the phenomena of concurrent changes in both perfusion and the blood oxygenation level dependence (BOLD) contrast [19, 20] during either physiology perturbation (e.g., brain stimulation) or pathology perturbation (e.g., hypoxia via ischemia); and these changes could affect the MRI intensity via either T_1_^app^ based mechanism owing to perfusion change or the transverse (or apparent transverse) relaxation time (T_2_/T_2_*) based mechanism owing to BOLD contrast.

To address these issues, we conducted a study aiming to: i) develop a robust neuroimaging approach to simultaneously measure and image the CBF change and the BOLD contrast by using the saturation-recovery (SR)-T_1_ MRI method; ii) validate this approach by conducting simultaneous *in vivo* measurements of CBF change using the SR-T_1_ MRI method and the relative CBF change using laser Doppler flowmetry (LDF) recording under transient hypercapnia (increasing CBF) and acute ischemia (reducing CBF) conditions using a rat model at 9.4T; iii) investigate the effect of brain temperature change on T_1_^app^ and the apparent longitudinal relaxation rate (R_1_^app^ = 1/T_1_^app^) after the induction of hypercapnia or ischemia (see S1 Supporting Information); iv) establish the quantitative relationship between the CBF-sensitive R_1_ change (ΔR_1_^CBF^) and the CBF change (ΔCBF); and v) quantitatively study the temporal relationships among R_1_^CBF^ change, ΔCBF, relative CBF change and BOLD during and after hypercapnia or ischemia.

## Method and Theory

### The SR-T_1_ MRI pulse sequence for imaging T_1_^app^

**Fig 1:**
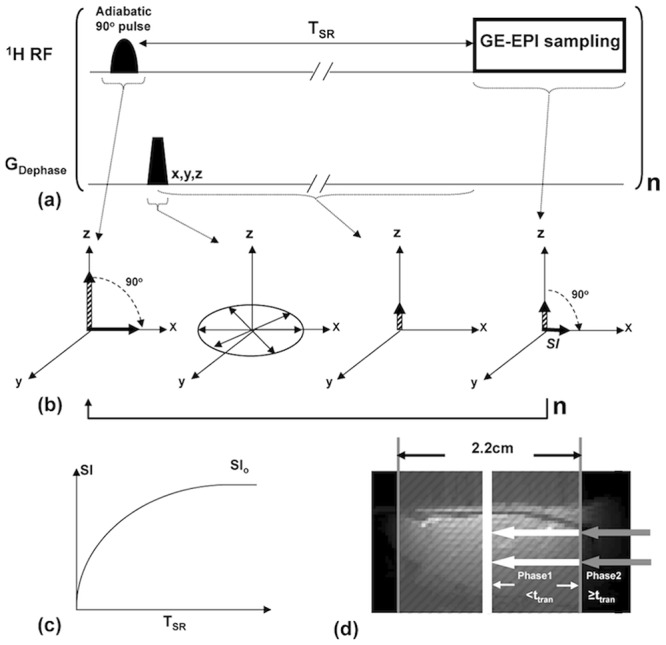
Schematic diagram of the SR-T_1_ MRI sequence and spin magnetization evolution in rotating frame. (a) Gradient-echo EPI (GE-EPI) sampling is applied after the saturation-recovery waiting time (T_SR_). There is no an extra delay time between the imaging acquisition and the next saturation pulse. This imaging measurement is repeated n times with varied T_SR_. (b) Schematic diagram illustrating the principle underlying the imaging sequence in the spin-rotating frame. It shows the rotation of longitudinal magnetization (M_z_), dephasing of transverse magnetization (M_xy_) and its evolution after T_SR_ and spin excitation pulse followed by GE-EPI acquisition. (c) Exponential recovery curve of GE-EPI signal intensity (SI) as a function of T_SR_. The regression of this curve determines the apparent T_1_ (T_1_^app^) value that is sensitive to perfusion. (d) Schematic diagram of two-phase model incorporated with the SR-T_1_ method developed in this study. The regional saturation zone (gray shaded area) achieved by a surface RF coil overlapped on a rat brain sagittal anatomic image. The white arrows stand for the Phase 1 arterial spins in the saturated region traveling into the image slice within the time window ttran (i.e., when T_SR_<t_tran_) The gray arrows indicate the unsaturated Phase 2 arterial blood spins flowing into the image slice when the traveling time is longer than t_tran_ or equal to (i.e., when T_SR_ ≥ t_tran_).

The MRI perfusion method described herein relies on quantitative T_1_^app^ mapping using the magnetization saturation-recovery preparation without slice selection and fast gradient-echo echo-planar imaging (GE-EPI) sampling [21]. The regional saturation-recovery preparation is confined by using a RF surface coil with a focal, intense RF field (B_1_) covering the rat brain [22]. Fig 1 is the schematic diagram showing the imaging pulse sequence and principle underlying the SR-T_1_ MRI method. The magnetization saturation preparation is achieved by an adiabatic half passage 90° RF pulse, which is insensitive to the inhomogeneous B_1_ field of a surface coil, immediately followed by dephasing gradients (G_Dephase_) in three dimensions (Fig 1a). The adiabatic 90° pulse rotates the longitudinal magnetization (M_z_) into the transverse plane in a spin-rotating frame, resulting in the transverse magnetization (M_xy_) with the same magnitude of M_z_. The M_xy_ rapidly loses its phase coherence because of the strong dephasing effect by G_Dephase_. The overall effect of the magnetization saturation preparation in combination with the dephasing gradients is to approach zero magnetizations for both M_z_ and M_xy_ components (Fig 1b). This magnetization preparation is independent of the initial M_z_ value or its reduction from the previous scan owing to the partial magnetization saturation effect when a relatively short TR is applied. Therefore, no extra delay before the adiabatic 90° pulse is needed. This feature can significantly shorten the total image acquisition time and improve the temporal resolution for imaging T_1_^app^ as compared to the conventional inversion-recovery preparation in which the net magnitude of inverted M_z_ depends on the initial M_z_ prior to the inversion pulse and the effect of partial saturation as a function of TR, thus, a relatively long TR is preferred for each T_IR_ measurement. During the period of saturation-recovery time (T_SR_), the longitudinal magnetization starts to relax and recover approximately according to an exponential function (Fig 1c). This recovered M_z_ after a period of T_SR_ is rotated to the transverse plane again by a spin excitation RF pulse with a nominal 90° flip angle, and then sampled by EPI acquisition. The detected EPI signal intensity (SI) without considering the perfusion effect on T_1_^app^ obeys the following equation:

$$SI=SI_{0}\cdot [1-e^{\left(-\frac{T_{SR}}{T_{1}^{app}}\right)}]\cdot e^{\left(-\frac{TE}{T_{2}^{*}}\right)}$$ (1)

where TE is the spin echo time; and T_2_* is the apparent transverse relaxation time, which is sensitive to magnetic field inhomogeneity and susceptibility effect, such as the BOLD contrast; SI_0_ is the EPI signal intensity when TE = 0 and T_SR_ = ∞. This equation can be used for T_1_^app^ regression based on a number of SI measurements with varied T_SR_. When T_SR_ is sufficiently long (e.g., ≥5T_1_^app^ as applied in this study) and TE>0, the second term in (Eq 1) approaches one, and (Eq 1) becomes:

$$SI^{*}=SI_{0}\cdot e^{\left(-\frac{TE}{T_{2}^{*}}\right)}$$ (2)

Under this condition, SI* is determined by the T_2_* relaxation process and becomes independent of T_1_^app^; thus, this signal can be used to quantify the “true” BOLD contrast without confounding effect from the saturation effect caused by the perfusion contribution [19, 20, 23]. Moreover, the addition of SI* measurement with a long T_SR_, thus, a long TR is also critical to improve the reliability and accuracy for T_1_^app^ regression, which is essential in determining the absolute CBF change, though it leads to a relatively low temporal resolution for simultaneously obtaining T_1_^app^ and BOLD images.

In this study, two brain conditions are defined by the subscript “RC” standing for the Reference Condition (i.e., control) before the induction of physiological/pathological perturbation, and the subscript “PC” standing for the Perturbed Condition by the induction of either hypercapnia (hyper-perfusion) or ischemia (hypo-perfusion). Accordingly, the BOLD contrast can be quantified by:

$$BOLD=\frac{SI_{PC}^{*}-SI_{RC}^{*}}{SI_{RC}^{*}}\;\;or\;\;rBOLD=\frac{SI_{PC}^{*}}{SI_{RC}^{*}}$$ (3)

where rBOLD stands for the relative BOLD.

### Two-phase arterial spin modeling of the SR-T_1_ MRI method

The Block equation describes the dynamic behavior of brain water magnetization as the following:

$$\frac{dM_{b}(t)}{dt}=\frac{M_{b}^{0}-M_{b}(t)}{T_{1}}+f[M_{a}(t)-M_{v}(t)]$$ (4)

where M_a_, M_b_ and M_v_ are the longitudinal water magnetization of the arterial blood, brain tissue and venous blood respectively; M_b_^0^ is the equilibrium value of M_b_; T_1_ is the brain tissue water longitudinal relaxation time in the absence of blood flow; *f* represents the CBF value. Associated with the proposed experimental MR preparation and acquisition, we solve (Eq 4) with a two-phase arterial spin model as illustrated in Fig 1d, showing the schematic graph of the global brain region (shaded gray area) saturated by the RF coil overlapped on a rat brain sagittal anatomic image. Phase 1 represents the time window in which the image slice receives the flowing saturated arterial spins within the regional saturation region (as indicated with the white arrows in the Fig 1d); and Phase 2 characterizes the time window when the fresh, fully relaxed arterial spins sitting outside of the saturation region (as indicated with the gray arrows in Fig 1d) flowing into the image slice. Assuming the arterial blood spins travel smoothly as bulk flow at a constant speed without any turbulence, the fresh spins at the edge of the saturation region will take the artery transit time (t_tran_) to reach the image slice. For the SR-T_1_ measurement with the boundary condition of M_b_(t = 0) = 0, the final solution (see S1 Supporting Information for details) for (Eq 4) and Phase 1 when t< t_tran_ follows:

$$\begin{array}{l} M_{b}(0-t_{tran})=M_{b}^{0}\left[\left(1-e^{-\frac{t}{T_{1}^{app}}}\right)-\frac{f}{\lambda \cdot (\frac{1}{T_{1}^{app}}-\frac{1}{T_{1a}})}\left(e^{-\frac{t}{T_{1a}}}-e^{-\frac{t}{T_{1}^{app}}}\right)\right]=M_{b}^{0}(A-B)\\ A=1-e^{-\frac{t}{T_{1}^{app}}}\\ B=\frac{f}{\lambda \cdot (\frac{1}{T_{1}^{app}}-\frac{1}{T_{1a}})}\left(e^{-\frac{t}{T_{1a}}}-e^{-\frac{t}{T_{1}^{app}}}\right) \end{array}$$ (5)

where T_1a_ is the longitudinal relaxation time of arterial blood and

$$\frac{1}{T_{1}^{app}}=\frac{1}{T_{1}}+\frac{1}{T_{1}^{temp}}+\frac{f}{\lambda }\;\;or\;\;R_{1}^{app}=R_{1}+R_{1}^{temp}+\frac{f}{\lambda }$$ (6)

where λ (= 0.9 ml/g) is the brain-blood partition coefficient, T_1_^temp^ (R_1_^temp^) is the contribution of temperature-dependent longitudinal relaxation time (rate) caused by brain temperature alteration during physiological or pathological perturbation (see S1 Supporting Information).

During Phase 2 the fully relaxed artery spins in the blood outside the saturation region flow into the rat brain, and will approach the image plane and exchange with the brain tissue water spins when t≥t_tran_. Final solution for Phase 2 of (Eq 4) when t≥t_tran_ with the initial condition using (Eq 5) with the boundary condition of t = t_tran_ gives:

$$M_{b}(t\geq t_{tran})=M_{b}^{0}\left[1-e^{-\frac{t}{T_{1}^{app}}}\cdot (1-C_{A}+C_{B})\right]$$ (7)

where C_A_ and C_B_ are the constants which equal to A and B in (Eq 5), respectively, when t = t_tran_, they reflect the boundary condition and ensure the function continuity between Phase 1 and Phase 2. Therefore, when the saturation recovery time is shorter than t_tran_ (Phase 1), the magnetization of brain tissue water relaxes following (Eq 5) whereas when the saturation recovery time is longer than or equal to t_tran_ it will relax according to (Eq 7) instead. It is clear that the brain magnetization recovery with a long saturation recovery time of T_SR_≥t_tran_ (Phase 2) follows a single exponential relaxation time T_1_^app^ (Eq (7)), while the magnetization recovery in Phase 1 (T_SR_<t_tran_) in theory is influenced by both T_1_^app^ and T_1a_ (see (Eq 5)).

Close examination of (Eq 5) for Phase 1, one can see that the signal recovery described by the term A only depends on T_1_^app^ whereas the signal recovery of the term B relies on both T_1_^app^ and T_1a_. A simulation study was conducted using (Eq 5) and the parameters relevant to this study for comparing the relative contributions of A and B terms (see S1 Supporting Information) and it turns out that the term B in (Eq 5) is less than 4% of the term A within a reasonable artery transit time range (100–500ms) in the rat brain [1, 24–26]. Therefore, a single exponential recovery according to the term A is a rational approximation for the rat brain application during Phase 1 (T_SR_<t_tran_) since the magnetization contribution from the term B is negligible. Therefore, the exponential recovery functions for both Phase 1 and Phase 2 can be unified as a single exponential recovery function according to T_1_^app^. In summary, a single exponential fitting of T_1_^app^ based on multiple SR-T_1_ MRI measurements with varied T_SR_ values presents a simple approach and good approximation for imaging T_1_^app^ or R_1_^app^ which can be quantitatively linked to CBF according to (Eq 6).

### Imaging T_1_^app^ (or R_1_^app^), T_1_^CBF^ (or R_1_^CBF^) and CBF change (ΔCBF)

The T_1_ and R_1_ term in (Eq 6) represent the intrinsic brain tissue property of longitudinal relaxation time and rate, respectively; they are usually insensitive to physiological changes and can be treated as constants. The R_1_^app^ difference between the reference and perturbed conditions becomes:

$$\Delta R_{1}^{app}=\Delta R_{1}{}^{temp}+\frac{\Delta CBF}{\lambda }$$ (8)

where ΔCBF = CBF_PC—_CBF_RC_. Thus, CBF change (ΔCBF) between perturbation and reference conditions can be calculated from the following equation:

$$\Delta CBF=\lambda \cdot (\Delta R_{1}{}^{app}-\Delta R_{1}{}^{temp})=\lambda \cdot \Delta R_{1}{}^{CBF}$$ (9)

where ΔR_1_^CBF^ presents the R_1_ change, which is solely attributed to the CBF change induced by physiopathological perturbation; and it can be imaged by the SR-T_1_ MRI method through three steps: i) image brain MRI SI as a function of T_SR_ during both control and perturbed conditions, and then determine the T_1_^app^ values in each image pixel by the exponential regression of measured SI as a function of T_SR_ according to (Eq 1); ii) subtract the control R_1_^app^ (= 1/T_1_^app^) value from the perturbed R_1_^app^ value resulting in ΔR_1_^app^; iii) determine ΔT_1_^temp^ or ΔR_1_^temp^ caused by a brain temperature change induced by perturbation (see S1 Supporting Information), then calculate ΔR_1_^CBF^ and ΔCBF according to (Eq 9). The unit of CBF in (Eq 9) is ml/g/second, which can be converted to a conventional unit of ml/g/min by multiplying CBF by 60.

## Materials and MRI Measurements

### Animal preparation and Experiment Design

All animal experiments were conducted according to the National Research Council’s Guide for the Care and Use of Laboratory Animals and under the protocols approved by the Institutional Animal Care and Use Committee of University of Minnesota. Twelve male Sprague-Dawley rats weighing 328 ± 35 g were included in this study. The rat was initially anesthetized and intubated using 5% (v/v) isoflurane in N_2_O:O_2_ (60/40) gas mixture. Both femoral arteries and left femoral vein were catheterized for physiological monitoring and blood sampling. Five rats were used for simultaneous MRI/LDF/temperature measurements. The LDF/Temperature instrument (Oxford Optronix, UK) was used to concurrently measure the percentage change of CBF or the relative CBF change that is defined as rCBF = CBF_PC_/CBF_RC_ and the brain temperature change in the cortical region in one hemisphere by inserting the LDF/Temperature probe (0.5 mm diameter) into the brain tissue through a small hole (3×3 mm^2^) passing both skull and dura (1.5–4 mm lateral, 1.5–3 mm posterior to the bregma, 1.9 mm deep). The soft tissue around the hole was kept to minimize magnetic susceptibility artifacts in MRI. After the surgical operation, the rat was placed in a home-built cradle incorporating ear bars and a bite bar to reduce head movement and to ensure proper positioning inside the MRI scanner. The animal anesthesia was maintained at 2% isoflurane. Rectal temperature was maintained at 37.0±0.5°C by a circulating/heating water blanket and the rate and volume of ventilation were adjusted to maintain normal blood gases.

Mild transient hypercapnia was induced in eight of the twelve rats used in this study by ventilating the gas mixture of 10% CO_2_, 2% isoflurane and 88% N_2_O:O_2_ (60/40) for 7 minutes; three of the eight rats were used for simultaneous measurements of ΔR_1_^CBF^, ΔCBF and BOLD using the SR-T_1_ MRI method, and rCBF and temperature (T) change using the LDF/Temperature probe; and other five rats were used to conduct the MRI experiments only.

All twelve rats performed 1-minute occlusion of the two carotid arteries to achieve acute, global brain ischemia using the four-blood-vessel-occlusion rat model [27]. The transient hypercapnia experiment was performed first, followed by the acute ischemia experiment, and the rats were sacrificed by KCl injection for approaching cardiac arrest at the end of the experiment. There was an adequately long waiting time between these studies to ensure stable animal conditions prior to each perturbation and measurement. The SR-T_1_ GE-EPI data were acquired for two minutes prior to each perturbation of transient hypercapnia (7 minutes), acute ischemia (1 minute) or KCl injection for approaching cardiac arrest. This control (or prior-perturbation) imaging acquisition period is defined as Stage 1. The duration during either the transient hypercapnia or acute ischemia perturbation is defined as the perturbation stage or Stage 2. Finally, the relatively long post-perturbation period was divided into three stages (i.e., early Stage 3; middle Stage 4 and late Stage 5).

### MRI measurement

All MRI experiments were conducted on a 9.4T horizontal animal magnet (Magnex Scientific, Abingdon, UK) interfaced to a Varian INOVA console (Varian, Palo Alto, CA, USA). A butterfly-shape ^1^H surface coil (2.8×2.0 cm with the short axis paralleled to the animal spine) was used to collect all MRI data. Scout images were acquired using a turbo fast low angle shot (TurboFLASH) imaging sequence [28] with the following acquisition parameters: TR = 10 ms, TE = 4 ms, image slice thickness = 2 mm, field of view (FOV) = 3.2 cm×3.2 cm; image matrix size = 128×128.

The magnetization saturation of water spin inside the rat brain was achieved by using the local B_1_ field of the RF surface coil and the adiabatic 90°RF pulse followed by three orthogonal dephasing gradients. GE-EPI (TE = 21 ms; FOV = 3.2cm×3.2cm; image matrix size = 64×64; single slice coronal image with 2 mm thickness) combined with the saturation-recovery preparation was used to image T_1_^app^ with seven T_SR_ values of 0.004, 0.1, 0.2, 0.3, 0.4, 0.5 and 10 s, which resulted in a temporal resolution of 11.9 s for obtaining one set of T_1_^app^ and BOLD images. This SR-T_1_ GE-EPI imaging sequence (see Fig 1a) was applied to: i) measure ΔR_1_^CBF^ resulting from either hypercapnia (CBF increase) or acute ischemia (CBF reduction) compared to the control condition; ii) determine the relationship between brain temperature change and ΔR_1_^temp^ immediately after the cardiac arrest (i.e., CBF = 0) with a KCl bolus injection (see S1 Supporting Information for details); and iii) determine ΔCBF values and then compare and correlate the values with the LDF measurement results.

### Data analysis

MRI data analysis was performed using the STIMULATE software package (Stimulate, Center for Magnetic Resonance Research, University of Minnesota, USA) [29] and the Matlab software package (The Mathworks Inc., Natick, MA, USA). LDF data was sub-sampled to match the corresponding MRI sampling rate and processed with home-written Matlab programs. Both region of interest (ROI) and single pixel MRI data taken from the rat sensory cortical region were used to perform the T_1_^app^ regression analysis and to determine ΔR_1_^app^, ΔR_1_^temp^ (see S1 Supporting Information for details) and ΔCBF according to Eqs (8) and (9). The least-square nonlinear curve-fitting program using the Matlab software was applied to perform the T_1_^app^ regression analysis. The regression accuracy was estimated by the sum squared error (sse) and the square of regression coefficient (R^2^).

To improve the quantification accuracy, the GE-EPI data were averaged within each stage as defined above and then applied to calculate the averaged values of ΔR_1_^app^, ΔR_1_^temp^ and ΔCBF based on the transient hypercapnia or acute ischemia measurement. The reference control CBF (CBF_RC_) was further estimated from the averaged ΔCBF values and its corresponding relative CBF changes (rCBF) measured by LDF under ischemia condition and during the reperfusion period after the acute ischemia. ROIs (ranging from 24 to 52 pixels) were chosen from the cortical brain region in the intact hemisphere with the location being approximately contralateral to the LDF recording side for those experiments performing simultaneous MRI and LDF/Temperature measurements in order to avoid the MRI susceptibility artifacts caused by the LDF/Temperature probe. The GE-EPI data acquired with the longest T_SR_ of 10 s (i.e., ≈ 5T_1_) were used to calculate BOLD according to (Eq 3).

The R_1_^app^ images at the control stage and a series of ΔCBF and BOLD images measured during and post hypercapnia and/or ischemia stages were created on a pixel-by-pixel basis (pixel size 0.25×0.25×2 mm^3^, with nearest neighbor interpolation) with two-dimensional median filtering and then overlapped on the anatomic image. Paired t-test was applied to compare the T_1_^app^ values measured at reference and perturbation conditions obtained from either ROIs or single pixel, as well as to compare the regressed T_1_^app^ values using ROI or single pixel data under a given condition. A p value of < 0.05 is considered as statistically significant.

## Results

### Reliability and sensitivity of T_1_^app^ measurement using the SR-T_1_ MRI method

**Fig 2:**
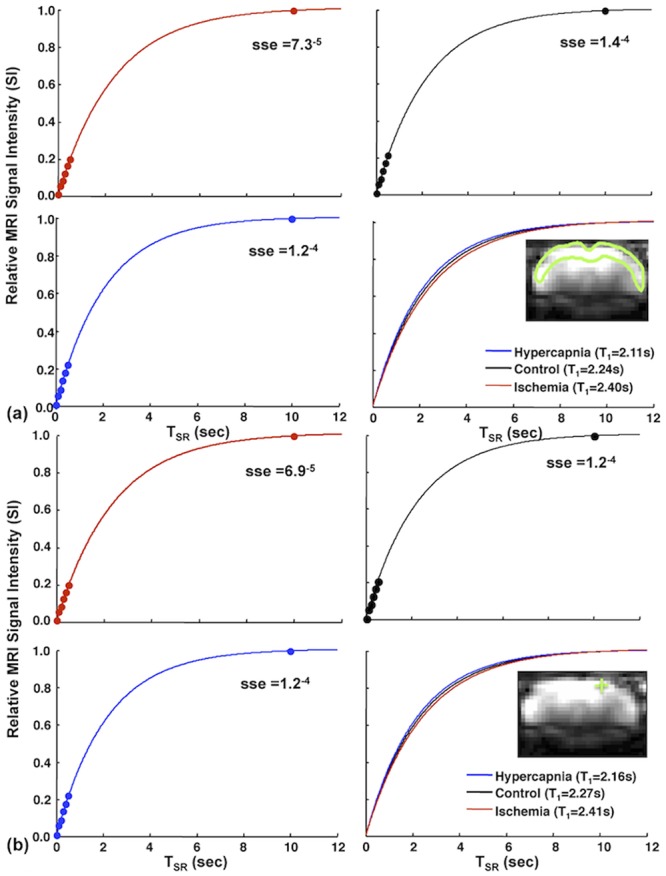
T_1_^app^ regression time courses with the SR-T_1_ MRI method. Time courses from a single SR-T_1_ GE-EPI measurement and T_1_^app^ regression under control (black lines); hypercapnia (blue lines); and ischemia (red lines) conditions based on (a) ROI and (b) single EPI pixel located inside the rat brain cortex. Colored dots are the signal data points imaged at different T_SR_s under different conditions. Three T_1_ fitting lines and their T_1_^app^ values under three conditions are also displayed in (a) and (b). The inserts show a coronal brain GE-EPI from a representative rat, indicating the location of (a) the ROI (enclosed in the green solid line) and (b) a single pixel (green cross mark) used for the regression (sse stands for the sum squared error; R^2^ = 0.99).

The averaged T_1_^app^ value measured using the SR-T_1_ MRI method in the rat cortex region under the normal physiological condition was 2.30±0.03 s (n = 12) at 9.4T. Fig 2 demonstrates a representative, single SR-T_1_ GE-EPI measurement under control, hypercapnia and ischemia condition, respectively, and the T_1_^app^ regression results based on ROI (Fig 2a) and single pixel (Fig 2b) data analysis without signal averaging. All the experimental data fitted well with an exponential function (R^2^≥0.99 and sse <2×10^-4^). The T_1_^app^ regression curves and the fitted T_1_^app^ values measured under control, hypercapnia and ischemia conditions were distinguishable and highly reproducible; and the results between the ROI and single pixel data analysis were consistent (Fig 2). For instance, no statistical difference was found between the T_1_^app^ values obtained from the ROI analysis versus single pixel analysis under either the hypercapnia (p = 0.93, 12 image volumes using paired t-test) or the ischemia (p = 0.83, 5 image volumes using paired t-test) condition. These results reveal a high reliability of the proposed MRI method for imaging T_1_^app^ and its change down to the pixel level, and this reproducibility is crucial in generating reliable T_1_^app^ maps. Moreover, the determined T_1_^app^ values under the hypercapnia and ischemia perturbations were statistically different from the control T_1_^app^ value (p<0.01), indicating that the T_1_^app^ relaxation process is sensitive to the perfusion changes induced by physiology/pathology perturbations. It is worth to note that adding more T_SR_ points in a median T_SR_ range (e.g., few seconds) could be helpful to further improve the fitting accuracy of T_1_^app^ (or R_1_^app^) measurement with a tradeoff of reduced imaging temporal resolution; nevertheless, the benefit on the outcome of ΔR_1_^app^, thus, ΔCBF measurement is insignificant because of the cancelation of the systematic errors of R_1_^app^ measurements under control and perturbation conditions (data not shown herein). One could optimize the T_SR_ values and the number of T_SR_ points for achieving a proper balance between the T_1_^app^ fitting accuracy and imaging temporal resolution.

### Relationships between rCBF, relative R_1_^CBF^ and BOLD induced by perturbations

**Fig 3:**
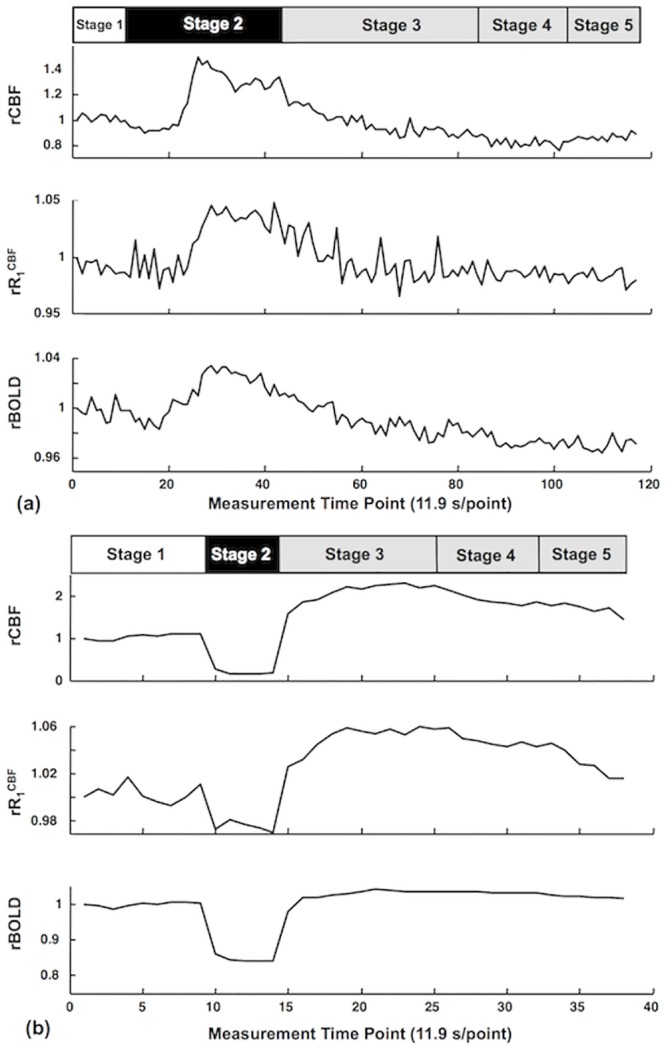
Time courses of relative rR_1_^CBF^, rBOLD and rCBF of hypercapnia and global ischemia experiments. Time courses of relative R_1_^CBF^ (rR_1_^CBF^) and relative BOLD (rBOLD) measured by the SR-T_1_ MRI method, relative CBF change (rCBF) measured by LDF before, during and after (a) hypercapnia and (b) ischemia perturbation from a representative rat (data extracted from a region of interest). The bar graphs on top indicate the experimental acquisition protocol of (a) hypercapnia and (b) ischemia. Five stages are defined for imaging acquisition under varied animal conditions. Stage 1 represents the control (or prior-perturbation) period (2 minutes) prior to the induction of perturbations (i.e., hypercapnia or ischemia). Stage 2 represents the perturbation period either for the transient hypercapnia (7 minutes) or acute ischemia (1 minute). Stages 3, 4 and 5 represent the three post-perturbation periods with varied time duration after either the transient hypercapnia or acute ischemia perturbation. Because the post-perturbation effects on CBF and BOLD responses were much shorter for the 1-minute acute ischemia perturbation than that of 7-minute transient hypercapnia perturbation, the durations for these three stages were different for these two perturbation studies: i) for the hypercapnia perturbation: 8 minutes for Stage 3; 3.4 minutes for Stage 4 and 3.2 minutes for Stage 5; and ii) for the ischemia: 2.4 minutes for Stage 3; 1.4 minutes for Stage 4 and 1.2 minutes for Stage 3. Please note that there were noticeable dynamic response delays (about 2 minutes) of CBF and BOLD response owing to a large dead volume in the ventilation system when slowly increasing the inhaled CO_2_ concentration during the hypercapnia experiment.

Fig 3 shows the high temporal resolution (~12 s per data point) time courses of relative R_1_^CBF^ (rR_1_^CBF^: the R_1_^CBF^ ratio between RC and PC conditions) and rBOLD measured by the SR-T_1_ MRI method from the ROI located in the rat sensory cortex and rCBF measured by LDF in the similar brain region before, during and after (a) transient hypercapnia and (b) acute ischemia perturbation from a representative rat. Despite some fluctuations, these time courses display expected temporal behaviors and dynamics. First, there are approximately parallel trends among all of the measured time courses. Secondly, the transient hypercapnia led to significant increases in the measured parameters owing to the vascular dilation effect, thus, increasing perfusion followed by a recovery back to the baseline level after the termination of hypercapnia. Thirdly, the acute ischemia caused rapid reductions in all measured parameters followed by a substantial overshooting (reperfusion) after the termination of ischemia and a slow recovery to the baseline level. Nevertheless, a careful examination of Fig 3 suggests a stronger temporal correlation between the measured rR_1_^CBF^ and rCBF (correlation coefficient = 0.84 for hypercapnia and 0.90 for ischemia, p<0.01) compared to the correlation of rR_1_^CBF^ versus rBOLD (correlation coefficient = 0.78 for hypercapnia and 0.75 for ischemia, p<0.01). Relative BOLD (rBOLD) shows a more significant undershoot after the hypercapnia (Fig 3a) and a smaller overshooting after the ischemia than that of rR_1_^CBF^ and rCBF (Fig 3b). These results reveal the feasibility of the SR-T_1_ MRI method to simultaneously measure BOLD and rR_1_^CBF^ which is tightly correlated to the CBF change; and its ability to temporally dissociate the BOLD and CBF responses with relatively high temporal resolution. It is also interesting to note that the rR_1_^CBF^ time course had a relatively large fluctuation compared to the BOLD time course, presumably owing to a moderately low inherent contrast-to-noise ratio to measure the CBF change.

During the ischemia perturbation, R_1_^CBF^ decreased 4.7±1.2% (n = 5) compared to the control condition, which was equivalent to a 5.1±1.4% increase of ΔT_1_^CBF^; accordingly, CBF decreased 89.5±1.8% and BOLD decreased 23.1±2.8%. During the hypercapnia, in contrast, R_1_^CBF^ increased 5.1±0.8% (n = 3); ΔT_1_^CBF^ decreased 4.8 ± 0.7%; CBF increased 82±12%; and BOLD increased 4.5±2.7%.

**Fig 4:**
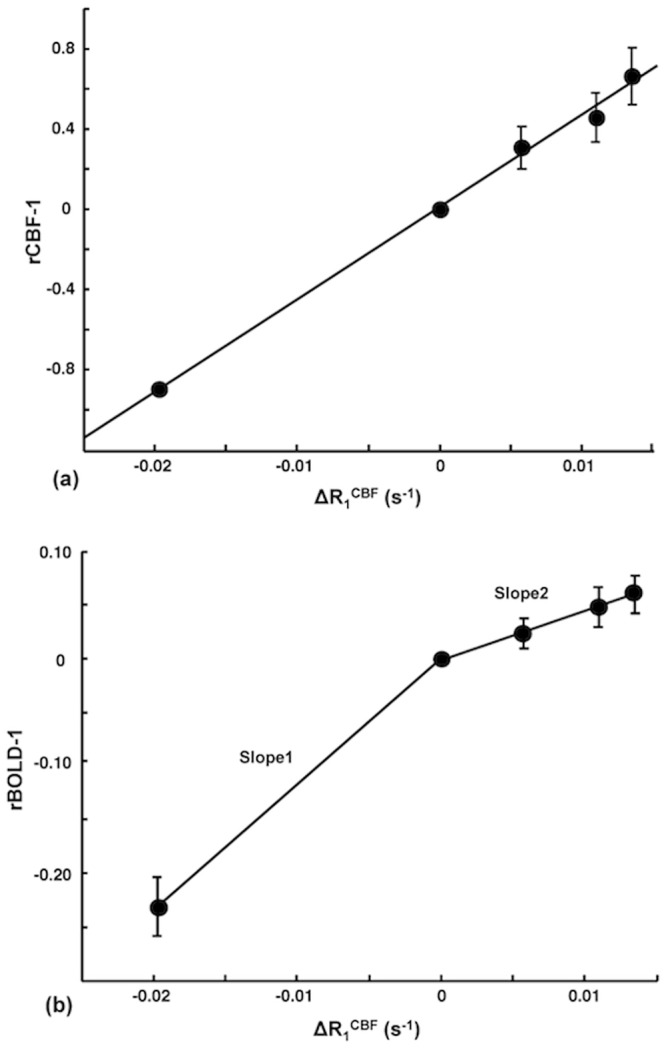
Correlation between ΔR_1_^CBF^ versus (a) rCBF-1 and (b) rBOLD-1. Correlation between the averaged ΔR_1_^CBF^ measured by the SR-T_1_ MRI method versus (a) rCBF-1 and (b) rBOLD-1 obtained during the five stages of ischemia experiment. The vertical bars indicate the standard error (SEM) (n = 5). There is a strong, positive correlation between (rCBF-1) and ΔR_1_^CBF^ in (a). In contrast, there are two distinct linear fitting slopes between (rBOLD-1) and ΔR_1_^CBF^ in (b) owing to the decoupled change between them during the post-ischemia stages.

Fig 4 shows the plots of ΔR_1_^CBF^ versus (rCBF-1) (Fig 4a) and versus (rBOLD-1) (Fig 4b) measured for the ischemia study. It indicates an excellent consistency and strong linear correlation between the CBF change and ΔR_1_^CBF^ across all five stages studied; and the correlation can be described by the following numerical equation that was obtained by the linear regression:

$$(rCBF-1)=45.9\cdot \Delta R_{1}^{CBF}+0.01$$ (10)

with R^2^ = 0.99. In contrast, two distinct linear fitting slopes (slope ratio = 2.6) were observed between ΔR_1_^CBF^ and (rBOLD-1), indicating the independence of the SR-T_1_ MRI method for simultaneously determining ΔR_1_^CBF^ and BOLD; and showing the decoupled changes of these two physiological parameters during the post-ischemia stages.

Table 1 summarizes the results of simultaneous rCBF (by LDF) and ΔR_1_^CBF^ measurements (by the SR-T_1_ MRI method) during the ischemia (Stage 2) and the first post-ischemia period (Stage 3) for each rat and averages among inter-subjects. The ΔR_1_^CBF^ value was used to calculate the CBF change (ΔCBF) according to (Eq 9), and then rCBF and ΔCBF were applied to estimate the reference control (or baseline condition) CBF (i.e., CBF_RC_) according to the following relationship:

$$CBF_{RC}=\Delta CBF/(rCBF-1)$$ (11)

**Table 1:** Summary of ΔR_1_^CBF^, ΔCBF, (rCBF-1) and the estimated reference control CBF.

	Ischemia Stage (Stage 2)				Ischemia Overshooting Stage (Stage 3)			
Rat No.	ΔR_1_^CBF^(s^-1^)	Calculated ΔCBF (ml/g/min)	rCBF-1	Control CBF (ml/g/min)	ΔR_1_^CBF^(s^-1^)	Calculated ΔCBF (ml/g/min)	rCBF-1	Control CBF (ml/g/min)
**1**	-0.023	-1.247	-0.883	1.413	0.014	0.751	0.332	2.263
**2**	-0.036	-1.928	-0.899	2.145	0.024	1.280	1.104	1.159
**3**	-0.014	-0.778	-0.924	0.841	0.016	0.880	0.597	1.474
**4**	-0.012	-0.664	-0.833	0.798	0.006	0.346	0.834	0.415
**5**	-0.013	-0.713	-0.938	0.760	0.007	0.389	0.445	0.874
**Mean ±SEM**	-0.020±0.005	-1.066±0.239	-0.895±0.018	1.191±0.267	0.013±0.003	0.729±0.172	0.662±0.139	1.237±0.310

Summary of ΔR_1_^CBF^, calculated CBF change (ΔCBF) based on ΔR_1_^CBF^, (rCBF-1) measured with LDF during the ischemia (Stage 2) and the first post-ischemia stage (Stage 3); and the estimated reference control CBF of five individual rats and its mean and standard error values.

The estimated CBF_RC_ was 1.19±0.27 ml/g/min calculated from the ischemia stage (Stage 2) data, and 1.24±0.31 ml/g/min calculated from the first post-ischemia stage (Stage 3) data, showing an excellent consistency between them. The estimated baseline CBF values in this study are coincident with the reported values in the literature ranging from 0.9 to 1.5 ml/g/min (1.29±0.05 ml/g/min) measured in the rat cortex under similar isoflurane anesthesia condition, which are summarized in S1 Supporting Information. Furthermore, if we approximate the small interception value of 0.01 to zero in (Eq 10) and replace (rCBF-1) term in (Eq 11) with the approximated (Eq 10), we derived CBF_RC_ = (60sec/min)·λ(ml/g)/45.9(sec) = 1.18 ml/g/min using the relationship of (Eq 9). This value based on the regressed slope of 45.9 sec in (Eq 10) using the data shown in Fig 4a is again in good agreement with the averaged literature value of 1.29±0.05 ml/g/min. These comparison results provide ample evidence supporting the feasibility and reliability of the proposed SR-T_1_ MRI method in measuring and quantifying the CBF changes induced by physiological/pathological perturbations.

**Fig 5:**
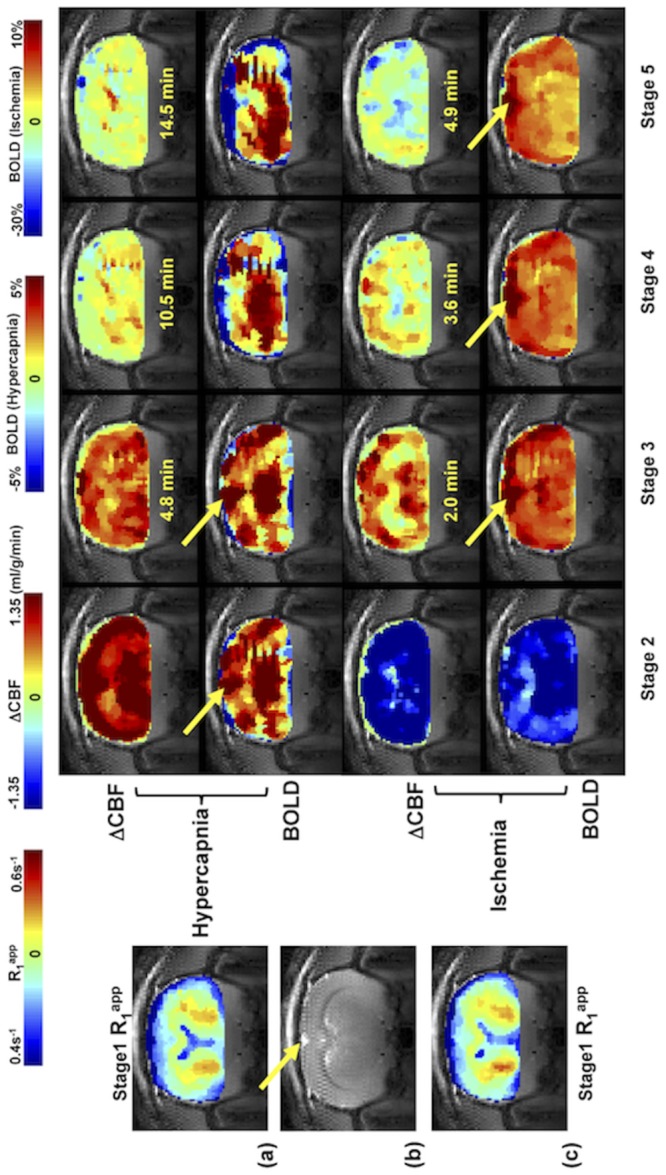
Coronal anatomic image, R_1_^app^ images, ΔCBF and BOLD images of a representative rat. (a) and (c) Reference control R_1_^app^ images (acquired during Stage 1); the SR-T_1_ MRI method generated ΔCBF and BOLD images of Stage 2 to Stage 5 obtained from (a) hypercapnia and (c) ischemia study, respectively from a representative rat brain. (b) Anatomic coronal image from the same rat. The time indicated between the ΔCBF and BOLD images is the approximate image sampling time after the termination of Stage 2. The yellow arrows point to the sinus vessel. The total image averaging time for Stages 2 to 5 were: 2.5, 6.5, 4.6 and 3.2 minutes for the hypercapnia study; and 1.0, 1.8, 1.4 and 1.2 minutes for the ischemia study.

Fig 5 shows the control R_1_^app^ images (coronal orientation) of rat brain, anatomic image, and the ΔCBF and BOLD images measured by the SR-T_1_ MRI method during and after the hypercapnia/ischemia perturbation in a representative rat without the use of LDF probe to avoid the susceptibility MRI artifacts. It illustrates that the SR-T_1_ MRI method is robust and sensitive for noninvasively imaging the CBF changes in response to a physiological (hypercapnia) or pathological (ischemia) challenge with a few minutes of image acquisition time. Moreover, the BOLD images can also be simultaneously obtained.

## Discussion

### Underlying mechanism for imaging CBF change in rat brain using the SR-T_1_ imaging method

Instead of focusing on the magnetization change by delivering tagged spins to the image slice(s) at a certain T_IR_ in the ASL approach, T_1_ perfusion model [2, 5, 6, 10] views CBF circulation as an enhanced longitudinal relaxation through T_1_^app^ as defined in (Eq 6). The rapid exchange between the saturated water protons in the image slice and the fully relaxed arterial blood water outside of the saturation region during the SR-T_1_ MRI measurement enables a quantitative link between T_1_^app^ and CBF. Based on the current MRI acquisition scheme used in this study, (Eq 5) (valid for Phase 1, when T_SR_ < t_tran_) and (Eq 7) (valid for Phase 2 when T_SR_ ≥ t_tran_) quantitatively describe the magnetization change as a function of T_SR_ for the SR-T_1_ MRI method with a two-phase perfusion model shown in Fig 1d. According to (Eq 5), the brain tissue relaxation depends on both T_1_^app^ and T_1a_ when T_SR_ < t_tran_ (Phase 1), however, the term B in (Eq 5) which contains T_1a_ accounts for only few percentages of the term A and its contribution to the magnetization relaxation becomes insignificant. Therefore, a single exponential recovery function according to the T_1_^app^ relaxation time provides a good approximation. When T_SR_ ≥ t_tran_ (Phase 2), the brain tissue magnetization relaxation solely follows T_1_^app^ according to (Eq 7). Therefore, T_1_^app^ dominates the magnetization change through the entire T_SR_ covering both Phase 1 and Phase 2, and can be robustly regressed for determining CBF changes. The excellent T_1_^app^ fitting curve (and excellent linearity in semi-log fitting, data not shown herein), as well as the high reproducibility and sensitivity to CBF alteration as shown in Fig 2 demonstrate that the single exponential function regression worked well in this study.

LDF measures a frequency shift in light reflected from moving red blood cells [30, 31]. It enables a real-time, continuous recording of relative (or percentage) CBF change in a focal region inside the brain; and is regarded as a standard tool for dynamic CBF measurements [32]. Moreover, LDF-based CBF measurements have been reported to be in good agreement with the radioactive microsphere CBF techniques [31, 33] as well as the hydrogen clearance CBF methods [34]. The excellent correlation between ΔR_1_^CBF^ measured with the SR-T_1_ method and relative CBF change recorded by LDF technique (Figs 3 and 4) during physiological/pathological perturbations, and the coincidence between the calculated baseline CBF results and the reported CBF values in the literature clearly suggest that ΔR_1_^CBF^ imaged by the SR-T_1_ MRI method quantitatively reflects the CBF changes. Besides the contributions from intrinsic T_1_ and CBF, T_1_^app^ can be also slightly influenced by other factors, for example, temperature. Although the temperature correction of T_1_^app^ could improve the accuracy of measurement, T_1_^app^ without the correction still provide a good approximation to calculate CBF change since the temperature induced T_1_ change is small (see S1 Supporting Information).

### Advantages, limitations and methodology aspects of the SR-T_1_ method for imaging CBF change and BOLD

The SR-T_1_ MRI method has several unique merits when comparing it with the conventional ASL techniques using an inversion-recovery preparation. First, the modeling used to quantitatively link T_1_^app^ and CBF in the SR-T_1_ MRI method is simple and it requires much less physiological parameters aiming to quantify absolute ΔCBF according to (Eq 9). Second, the SR-T_1_ MRI method relies on parametric T_1_^app^ mapping; thus, it does not require the paired control image as applied in most ASL methods for determining CBF change. Third, the saturation-recovery preparation avoids the requirement of relatively long TR constrained by the conventional T_1_ imaging methods based on the inversion-recovery preparation; this enables relatively rapid mapping of T_1_^app^ and ΔR_1_^app^ to generate the ΔCBF image as illustrated in Fig 5. Although the imaging of T_1_^app^ requires multiple measurements with varied T_SR_ values, its temporal resolution of 12 s per complete image set is comparable with other ASL methods (approximately 5–10 s). Fourth, the partial volume effect of cerebrospinal fluid (CSF) to ΔCBF is minimal because of the subtraction out the undisturbed CSF-related R_1_ contribution under various animal conditions, slow movement of CSF water spins (about 20 times slower than CBF, [35–37]) and the negligible exchange between CSF and brain tissue water spins.

This study was based on single slice measurement to prove the concept and feasibility of the proposed SR-T_1_ MRI method, nevertheless, it should be readily extended to multiple image slices covering a larger brain volume.

One technical limitation of the current SR-T_1_ MRI method is its inability to directly measure the baseline CBF value under a physiological condition of interest, thus, the control value of CBF_RC_ was indirectly estimated by two independent measurements of ΔCBF and rCBF using the SR-T_1_ MRI method and LDF, respectively, in this study. This technique is more useable for imaging CBF changes, thus, requiring two measurement conditions, for instance, reference control versus perturbation (similar to the BOLD measurement) as presented in this study.

A surface RF coil generates an inhomogeneous distribution of B_1_ in space, resulting in a non-uniform RF pulse flip angle for saturating the water magnetization if a linear RF pulse waveform is used. In this study, we applied an adiabatic half passage 90° RF pulse to achieve relatively uniform 90° rotation of M_z_ into the transverse plane and to improve saturation efficiency, in particular, in the brain cortical region where B_1_ is strong. The ROI for data processing was chosen from this region (see Fig 2 for an example). In the deep brain region, distant from the surface coil, it might not be warranted to approach an adiabatic 90° rotation if the RF power is inadequate in this brain region. As a result, the RF saturation efficiency (0≤α≤1) can drop in the deep brain region and lead to relatively low saturation efficiency. However, this imperfection, if it exists, should not cause a significant error in determining T_1_^app^ owing to the following two reasons.

First, the term of α was considered in the least square regression to calculate the T_1_^app^ value using the following formula

$$\begin{array}{l} SI=SI_{0}e^{-\frac{TE}{T_{2}^{*}}}(1-\alpha \cdot e^{-\frac{T_{SR}}{T_{1}^{app}}})\\ \;\;\;=k(1-\alpha \cdot e^{-\frac{T_{SR}}{T_{1}^{app}}}) \end{array}$$ (12)

in which three constants, *k*, α and T_1_^app^ are determined by regression. Though α can become less than 1 in the deep brain region if B_1_ strength is inadequately strong, it can be treated as a constant. The T_1_^app^ regression becomes insensitive to the absolute value of α; and the regression outcome is mainly determined by the exponential rate of SI recovery as a function of T_SR_.

Second, a nominal 90° excitation pulse and a very short TE were used in the GE-EPI sampling in this study. In addition, there was no extra delay between the EPI signal acquisition and the next RF saturation pulse. This configuration further reduces the residual M_z_ component (or suppresses the M_z_ recovery) before the next magnetization saturation preparation. It acts as extra magnetization saturation, resulting in an improved saturation efficiency and insensitivity of regression to the value of α. These notions are supported by the ΔCBF images (Fig 5a and 5c) showing relatively uniform CBF changes across the entire image slice including the deep brain region. In contrast, the BOLD images show the ‘hot’ spots around the sinus vein as pointed by the arrows in Fig 5 because of the large BOLD effect near a large sinus vein [19]. Such ‘hot’ spots were not observed in the ΔCBF images, indicating again that the ΔCBF image is more specific to the tissue perfusion and less susceptible to macro vessels. This differentiation between the simultaneously measured BOLD and ΔCBF images suggests that the SR-T_1_ MRI method indeed is able to independently but simultaneously measure two important physiological parameters: CBF change and BOLD. Although, there is a similar trend between the measured changes of ΔCBF and BOLD during ischemia or hypercapnia perturbation as shown in this study, there are clearly distinct characters in both spatial distribution and temporal behavior between the ΔCBF and BOLD images during the recovery periods after the perturbations, which provide complementary information of the brain hemodynamic changes in response to physiological/pathological perturbations.

The SR-T_1_ MRI method is based on the exponential fitting of R_1_^app^ using multiple EPI images with varied saturation-recovery times, and the R_1_^app^ changes caused by either hypercapnia or ischemia were small (<5%). Therefore, the accuracy of fitting is susceptible to the EPI image noise level, in particular, in the brain regions with EPI susceptibility artifacts or weak B_1_ of the surface coil.

### Relationship between ΔR_1_^CBF^ and the CBF change during ischemia and hypercapnia

It is known that the development of vasogenic edema usually occurs at a later phase, approximately 30 minutes after the induction of regional ischemia [38]; and the water accumulation in the ischemic tissue owing to cellular swelling takes place hours after the onset of ischemia [39, 40]. It is unlikely that vasogenic edema and water content change could be responsible for the measured ΔR_1_^CBF^ change in the present study since the global ischemia lasted only one minute and the SR-T_1_ MRI measurements were continued within a relative short period during the post-perturbation stages. Therefore, ΔR_1_^CBF^ imaged by the SR-T_1_ MRI method could be fully quantified to determine and image ΔCBF. This notion is evident from the results of the ischemia study; and it also holds true for the hypercapnia study. However, it would be interesting to investigate the longitudinal T_1_^app^ change in the severely or chronically ischemic brain region, which could be affected by the CBF change and possibly other physiopathological changes (infarction, edema, necrosis etc.) of brain tissue. Further study of their relative contributions to the T_1_^app^ change would be helpful to understand the evolution of the ischemic lesion and its relationship with CBF change.

A similar estimation using the relationship of rCBF and ΔCBF measured during the hypercapnia (Stage 2) resulted in the CBF_RC_ value of 1.36±0.35 ml/g/min (n = 3). This value is close to that were calculated with the data collected during the ischemic/post-ischemic stages (≈1.2 ml/g/min) although they are not exactly the same. This small discrepancy might be due to the slightly basal CBF drifting through the prolonged period of experiment because two experiments (hypercapnia and ischemia) were combined during the same MRI scanning session. It could also be related to the limited sampling size of hypercapnia experiments. Nevertheless, ΔCBF increase induced by hypercapnia calculated from ΔR_1_^CBF^ correlates well with rCBF measured by LDF.

### Correlation of R_1_^CBF^, CBF and BOLD during perturbations

Beside the aforementioned distinction of spatial responses between the ΔCBF and BOLD images owing to the different specificity to the hemodynamic response with varied vessel size, it is interesting to note that there were significantly mismatched temporal responses between measured ΔCBF and BOLD during both post-ischemia and post-hypercapnia stages. The “overshooting” effect was much less for BOLD than the ΔCBF and rCBF responses during the post-ischemia stage (Fig 3b), leading to two distinct slopes of linear regression between (rBOLD-1) and ΔR_1_^CBF^ (Fig 4b). There was also a substantial “undershooting” in the measured BOLD change during the later post-hypercapnia recovery stages (Fig 3a) compared to rCBF or rR_1_^CBF^. One explanation for this observation is that BOLD signal reflects a complex interplay among CBF, cerebral blood volume (CBV) and oxygen consumption rate (CMRO_2_) [19, 20]. Therefore, BOLD can become decoupled with the CBF change, and degree of the mismatched BOLD and ΔCBF relies on the fractional changes in CBF, CBV and CMRO_2_ in response to a particular perturbation. The quantitative interpretation of the mismatched rCBF-rBOLD behavior requires additional measurements of CBV and CMRO_2_, which is beyond the scope of this article. Nevertheless, this mismatch could be linked to the uncoupling between the metabolic and hemodynamic responses associated with a physiology or pathology perturbation, and it should be useful for indirectly estimating the CMRO_2_ time course during the perturbation if the CBV change can be measured independently or estimated using a sophisticated BOLD modeling (e.g., [41]). In addition, the measured BOLD using the SR-T_1_ MRI method under fully relaxed condition reflects the “true” BOLD without the CBF confounding effect; thus, further improve the outcome of quantification [23].

A local RF coil, such as a surface coil as used in the present study, has been commonly applied for most *in vivo* animal MRI/MRS brain studies. Its B_1_ field (or profile) induces regional longitudinal magnetization changes through saturation (or inversion) preparation prior to the EPI acquisition. The combination of regional M_z_ preparation and relatively short artery blood traveling time from the non-saturated region into the EPI slice in animal brains is the underlying mechanism for a quantitative link between ΔCBF and ΔR_1_ that can be robustly imaged by the SR-T_1_ MRI method. Consequently, the T_1_ or T_1_-weighted MRI signal changes commonly observed in the clinical imaging diagnosis, for instance, stroke patients, are at least partially attributed by the impaired perfusion, i.e., ΔCBF. Finally, the SR-T_1_ MRI method can also be combined with a volume RF coil for imaging ΔCBF with the implement of a slice-selective saturation preparation.

## Conclusion

In summary, we have described the SR-T_1_ MRI method for noninvasively and simultaneously imaging the absolute CBF change and BOLD in response to physiological/pathological perturbations. This imaging method was rigorously validated in the rat brain with simultaneous LDF measurements under global ischemia and hypercapnia conditions. It should provide a robust, quantitative MRI-based neuroimaging tool for simultaneously measuring the CBF change and BOLD contrast associated with physiological perturbations (e.g., brain activation) or pathological perturbations (e.g., stroke or pharmaceutical drug treatment).

## Supporting Information

S1 Supporting Information Detailed equation derivation for two-phase arterial spin model of the SR-T_1_ method and simulation results. Supporting information regarding the confounding effect of brain temperature change on T_1_^app^.
(DOCX)
