# Supplementary material for: Simultaneous Imaging of CBF Change and BOLD with Saturation-Recovery-T1 Method
Source: PLoS One. 2015 Apr 23;10(4):e0122563. doi: 10.1371/journal.pone.0122563 (PMC4408048; doi:10.1371/journal.pone.0122563)
Supplement: S1 Supporting Information — Detailed equation derivation for two-phase arterial spin model of the SR-T1 method and simulation results. Supporting information regarding the confounding effect of brain temperature change on T1 app. (DOCX) [file pone.0122563.s001.docx]

**S1 Supporting Information**

**THEORY, RESULTS and DISCUSSION**

**Supporting Information: Theory**

**Differential equation derivation for two-phase arterial spin model of the SR-T_1_ method and simulation results of B/A ratio in Eq. (6) as a function of arterial transit time** (**t_tran_)**

Assuming that the brain tissue is a single, well-mixed compartment, so that M_v_(t) = M_b_(t)/λ, λ is blood-tissue water partition coefficient (= 0.9 ml/g) [[1](#_ENREF_1)], Eq. (4) in the main body text can be rewritten as:

 (S.1)

The spins at the edge of the saturation region will take the arterial transit time (t_tran_) to reach the image slice. For the saturation-recovery (SR)-T_1_ measurement with a saturation-recovery time (t) being shorter than t_tran_ (t< t_tran_, i.e., Phase 1 in Fig. 1d), the magnetization recovery of the saturated arterial spin as a function of time (M_a_(t)) can be expressed as:

(S.2)

where T_1a_ is the longitudinal relaxation time of arterial blood; M_a_^0^ is the M_a_ value at an equilibrium state and can be approximated by M_a_^0^= M_b_^0^/λ. Eq. (S.2) has a solution,

(S.3)

Eqs. (S.1) and (S.3) lead to:

 (S.4)

Eq. (S.4) is one type of differentiation equations and can be solved with the following solution:

(S.5)

where C is a constant. For the SR-T_1_ experiment with the boundary condition of M_b_(t=0) = 0, the final solution for Phase 1 (t< t_tran_) of Eq. (S.1) or Eq. (4) becomes:

(5)

where

(6)

T_1_^temp^ (R_1_^temp^) is the contribution of temperature-dependent longitudinal relaxation time (rate) caused by brain temperature change associated with physiological or pathological perturbation. The fully relaxed arterial spins outside the saturation region in Phase 2 flow into the rat brain, and they will reach the image plane and exchange with the brain tissue water spins when t≥t_tran_. For the Phase 2 spins, M_a_= M_b_^0^/λ, therefore, Eq. (S.1) can be written as:

. (S.6)

Reorganize Eq. (S.6) and then combine it with Eq. (6) lead to:

 (S.7)

Solving Eq. (S.7) with the initial condition using Eq. (5) with t = t_tran_ gives:

(7)

where C_A_ and C_B_ are the constants which equal to A and B in Eq. (5), respectively, when t = t_tran_.

The difference between the blood and brain tissue longitudinal relaxation times is small at the magnetic field strength (B_0_) of 9.4T and the single-exponential recovery model provides a reasonable assumption. The C_A_ and C_B_ in the term (1-C_A_+C_B_) in Eq. 7 reflected the boundary condition between Phase 1 and Phase 2, and they were fitted intrinsically and accounted for non-linear least square regression. This simplification improves the accuracy of T_1_^app^ measurement. For instance, the averaged T_1_^app^ value based on the SR-T_1_ measurements in the rat brain cortex was 2.30 s in this study. The blood T_1_ (T_1_^blood^) can be estimated using an established equation of T_1_^blood^ = 0.129×B_0_+1.167 with the unit of second [[2](#_ENREF_2)], resulting in T_1_^blood^ ≈ 2.38 s at B_0_=9.4T. S1 Fig. shows the simulation results of B/A ratio as defined in Eq. (5) as a function of artery transit time and it demonstrates that term B is less than 4% of term A within a range of t_tran_ between 100 and 500ms in the rat brain. Therefore, the term B in Eq. 5 is negligible and a single exponential recovery according to the term A is a good approximation for the SR-T_1_ measurement.

**Confounding effect of brain temperature change on T_1_^app^ measurement**

It is known that the apparent longitudinal relaxation time (rate) T_1_^app^ (R_1_^app^) depends on temperature change [[3-6](#_ENREF_3)]. The transient hypercapnia or ischemia perturbation can induce a change not only in CBF but also in the brain temperature because of the temperature difference between the cardiac and brain bloods [[7](#_ENREF_7)]; both of these changes can contaminate the measured value of ∆R_1_^app^ (see Eq. (8)). Therefore, it is critical to find out how much R_1_ change is contributed from a temperature change during the CBF perturbation and how it affects the quantification of CBF change with the SR-T_1_ MRI method. The baseline CBF in the rat brain under normocapnia is 1.29 ml/g/min from Table S1 and blood tissue partition coefficient λ is 0.9ml/g, thus, the CBF related relaxation rate would be CBF/λ/60s=0.024s^-1^. The control R_1_^app^ value of rat brain cortex is 0.48 s^-1^ from our study, therefore, the CBF contribution to the R_1_^app^ value can be estimated by 0.024 s^-1^/0.48 s^-1^ = 5% and it only counts for several percent.

The quantitative relation between T_1_^app^ and temperature (T) can be described by:

 (S.8)

where T_1_^temp^ is the temperature-dependent longitudinal relaxation time; T_1_^’^ is the T_1_^temp^ value when T=∞; *E_a_* is the activation energy related to the T_1_ relaxation process, κ is the Boltzmann constant. It has been demonstrated that under normal physiological conditions there is an approximated relationship given by [[3](#_ENREF_3)]:

$T_{1,PC}^{temp}-T_{1,RC}^{temp}=m\cdot(T_{PC}-T_{RC})$ (S.9)

or

$\Delta T_{1}^{temp}=m\cdot\Delta T$ (S.10)

where the subscript “PC” and “RC” stands for Perturbation Condition and Reference Condition respectively; m is the tissue-specific T_1_^temp^ dependence coefficient on the brain temperature. Within a small range of brain temperature change induced by physiological or pathological perturbation, Eq. (S.10) can be further simplified as:

$\Delta R_{1}^{temp}\cong m^{'}\cdot\Delta T$ (S.11)

where m’ stands for a tissue-specific R_1_^temp^ dependence coefficient on the brain temperature change. This relationship and m’ can be determined through the regression of varied ∆T and ΔR_1_^temp^ values measured in the absence of perfusion (e.g., complete cardiac arrest with CBF=0). Consequently, the constant m’ can be used to determine ΔR_1_^temp^ during and after physiological/pathological perturbation if the brain temperature change caused by the perturbation can be measured. ΔR_1_^temp^ can be applied for further correction in order to precisely quantify ΔCBF with the SR-T_1_ MRI method.

**Supporting Information: Results**

**Animal physiology**

Physiological data of the experiment measured before the induction of perturbation and before KCl injection are summarized in Table S2. No significant changes in pCO_2_, pO_2_ and heart rate were observed. There was, however, a statistically significant decrease in pH and mean arterial blood pressure (MABP) between these two measurements.

**Temperature changes in rat brain during the perturbations and determination of tissue-specific T_1_^temp^ (m) or R_1_^temp^(m’)**

The maximal change of brain temperature increased 0.93±0.09ºC (n=3) during the transient hypercapnia perturbation; and decreased 0.26±0.02ºC (n=5) during the acute ischemia perturbation compared with the control condition before the induction of perturbation. S2a and S2b Figs. show the averaged changes of brain temperature induced by the hypercapnia and ischemia perturbation, respectively, measured during (Stage 2), and after the perturbation (Stages 3 to 5 as measured approximately around 4.8, 10.5 and 14.5 minutes after the termination of hypercapnia; and around 2.0, 3.6 and 4.9 minutes after the termination of ischemia, respectively) compared to the control condition (Stage 1). The averaging times for measuring the temperature for Stages 2 to 5 were about 2.5, 6.5, 4.6 and 3.2 minutes for the hypercapnia study; 1.0, 1.8 1.4 and 1.2 minutes for the ischemia study. The brain temperature increased significantly during all the post-perturbation stages as well as during the hypercapnia perturbation. In contrast, it dropped during the ischemia perturbation owing to the reduction of blood supply. These brain temperature changes led to the variation in R_1_ (i.e., ΔR_1_^temp^), which can be determined according to Eq. (S.11) if the constant of m’ can be determined.

The temperature dependence coefficient, m defined in Eq. (S.10) or m’ defined in Eq. (S.11), was measured in this study via quantifying the relationship between ∆R_1_^temp^ and its corresponding brain temperature change (ΔT) during the cardiac arrest induced by potassium chloride (KCl) bolus injection, in which ∆R_1_^temp^ was solely determined by ∆T. S2c Fig. illustrates one measurement result from a representative rat and the linear regression for calculating m’.

The averaged m was 36.1±8.7 (ms/ºC) and m’ was -0.007±0.002 (s^-1^/ºC) (R^2^ = 0.6±0.15; n=5). The ∆R_1_^temp^ values were calculated using the m’ constant and the four ∆T values measured during Stages 2 to 5 as illustrated in S2a and S2b Figs. Finally, ∆R_1_^temp^ values were used to calculate ∆R_1_^CBF^ and ΔCBF according to Eq. (9) in the main body text. The results indicate that the ∆T_1_^temp^ in the rat cortex could count for 15.3%, 3.2% and 19.9% of the contribution to the total ∆T_1_^app^ measured under the hypercapnia, ischemia and the first post-perturbation stage (i.e., Stage 3) after the ischemia perturbation, respectively.

**Confound effect of brain temperature change on T_1_^app^ measurement**

S3 Fig. depicts the high-temporal resolution (~12 s per data point without the averaging of repeatedly measured GE-EPI data) time courses of relative R_1_ (rR_1_ defined by R_1,PC_/R_1,RC_) measurements before the temperature correction (rR_1_^app^) and after the temperature correction (rR_1_^CBF^), showing a slight difference between them for both (a) hypercapnia and (b) ischemia studies. Therefore, the confound effect of brain temperature change was corrected in this study though it was small.

S4 Fig. summarizes the averaged results as measured during different stages. In this figure, the time course of rCBF measured by LDF serves as a reference that was used to compare its temporal tendency and correlation with the SR-T_1_ MRI results of rR_1_^app^ and rR_1_^CBF^ measured under the transient hypercapnia (S4a Fig.) and acute ischemia (S4b Fig.) perturbation. It is clearly evident that rCBF has a better temporal correlation with rR_1_^CBF^ after temperature correction than rR_1_^app^ across all post-perturbation stages. This is supported by the results of correlation coefficient (CC) analysis as summarized in Table S3. A slightly higher CC value was observed for the rR_1_^CBF^-rCBF time course correlation for all individual measurements compared to the rR_1_^app^-rCBF time course correlation, nevertheless the averaged CC values between the two comparisons did not reach statistical significance. These results indicate that correcting the effect of brain temperature change on the apparent T_1_^app^ rate can improve the correlation between the rCBF and rR_1_^CBF^ measurements. On the other hand, they also suggest that both rR_1_^app^ and rR_1_^CBF^ have a good correlation with rCBF and can rationally reflect the CBF change in response to perturbations, though rR_1_^CBF^ is more accurate than rR_1_^app^. This is not surprising owing to the fact that CBF dominates the change of T_1_^app^ or R_1_^app^; and temperature effect on T_1_^app^ or R_1_^app^ only accounts for less than 20% of ∆T_1_^app^ even the perturbations applied in this study resulted in substantially large brain temperature changes.

**Supporting Information: Discussion and Conclusion**

Two-phase arterial spin model provides the theoretical explanation of the SR-T_1_ method of imaging CBF change in this study. Saturation region achieved by the surface RF coil B_1_ field associated with the small size of the rat brain (and body) and relatively short arterial transit time makes a single exponential relaxation a good approximation of brain tissue T_1_^app^ measurement, which could be linked to the CBF change. The SR-T_1_ method could also be applied in human to image CBF change in response to functional brain stimulation [[8](#_ENREF_8)] and varieties of diseases although it will be more challenging than the animal model due to its low baseline CBF and likely small CBF change during stimulation when compared to the animal studies. Nevertheless, the SR-T_1_ method should enable an alternative, noninvasive and useful neuroimaging tool to investigate CBF change and BOLD under physiological and pathological conditions.

Though it is well documented that a rapid CBF change can lead to the brain temperature alteration and the water T_1_^app^ relaxation processing is sensitive to brain temperature, to our best knowledge, this study presents the first investigation aiming to quantify the brain temperature effects on both T_1_^app^ and CBF measurements during physiology/pathology conditions at 9.4T. This was accomplished through the simultaneous measurements of T_1_^app^/CBF/brain temperature changes during the course of physiological/pathological perturbation.

It has been reported that the blood T_1_ varies linearly with 1/T over the range of 0º-40º, suggesting a possible linear relationship between R_1_(1/T_1_) versus the brain temperature [[4](#_ENREF_4)]. Our results (e.g., S2c Fig.) are consistent with the relationship. The temperature dependent coefficient of m shown in Eq. (S.10) as measured in this study was 36.1 ± 8.7 (ms/ºC). This m value can be converted to 1.6% of T_1_^app^ per ºC, which is in excellent agreement with the value of ranging from 1.0% to 1.9% of T_1_^app^ per ºC reported in the literature [[9](#_ENREF_9), [10](#_ENREF_10)].

The effect of brain temperature change on T_1_^app^ and ∆CBF measurements depends on the type of perturbation. The rat brain temperature decreased 0.26 ± 0.02ºC during the ischemia perturbation; and its effect attributes only 3.2% of the total change in ∆T_1_^app^; in contrast, the maximum brain temperature increased 0.93 ± 0.09ºC and attributes ~15.3% of ∆T_1_^app^ during the hypercapnia perturbation. Similar to the hypercapnia, the temperature effect could also attribute 19.9 % of the measured ∆T_1_^app^ during the initial post-ischemia stage (Stage 3). Nevertheless, the perfusion change attributes more than 80% of the total T_1_^app^ change. As a result, the imaged ∆R_1_^app^ is dominated by ∆CBF and it provides a sensitive index reflecting the perfusion change in the brain tissue. This notion is confirmed by the statistical comparison results as summarized in Table S3, suggesting that both ∆R_1_^app^ and ∆R_1_^CBF^ have an excellent correlation with the absolute CBF changes in both ischemia and hypercapnia perturbations. Although ∆R_1_^CBF^ has a slightly better correlation with rCBF than ∆R_1_^app^, there was no statistically significant difference between them, presumably, a small confound effect of brain temperature change on the ∆R_1_^CBF^ values (<20%) could be approximately ignored, even when substantial brain temperature changes occurred during either the global ischemia or 10% CO_2_ hypercapnia perturbation. In addition, a strong and positive correlation is shown between (rCBF-1) and ∆R_1_^app^ during the whole ischemia experiment in S2d Fig. With the slope derived from S2d Fig., a baseline CBF value at reference condition can be estimated as CBF_RC_ = (60sec/min)·λ(ml/g)/ 52.3 (sec) = 1.03 ml/g/min using Eqs. (10) and (11) in main body text. This baseline CBF value is close to the value we obtained by employing R_1_^CBF^ and the literature reported values (Table S1). Therefore, for most physiological/pathological applications with technical difficulties in measuring the brain temperature change as well as the chronic disease with slow and small temperature change, the ∆R_1_^app^ image should provide a reasonable approximation reflecting the CBF change.

**Supporting Information: Tables**

**S1 Table. Summary of the literature cited CBF results.** Summary of the literature cited CBF results measured in the isoflurane-anesthetized rat under normal physiology condition.

| **Cited literature**  **Author, and year** | **Concentration of isoflurane (%)** | **Method of CBF measurement** | **CBF(ml/g/min) value at normal physiology condition** |
| --- | --- | --- | --- |
| Young et al, 1991 | 1.38 | autoradiography | 1.17 (cortex and subcortex) |
| Frietsch et al, 2000 | 1.2 | autoradiography | 1.3-1.4 (cortex and subcortex) |
| Machensen et al, 2000 | 1.4 | autoradiography | 1.5 (cortex) |
| Todd et al, 1996 | 1.6-1.8 | ^3^H-nicotine tracer | 1.37 (whole brain) |
| Hansen et al, 1988 | 1.2 | autoradiography | 1.47-1.54 (cortex) |
| Lenz et al, 1996 | 1.4 | autoradiography | 1.09-1.35 (whole brain) |
|  | 2.8 | autoradiography | 1.25-1.55 (whole brain) |
| Kim et al, 2007 | 1.3-1.5 | ASL | 1.5±0.18 (somatorsensory cortex) |
| Shen et al, 2005 | 1.1-1.2 | ASL | 1.2±0.7 (whole brain) |
| Sicard et al, 2005 | 1.15-1.25 | ASL | 1.10±0.04 (motor cortex) |
| Liu et al, 2004 | 1.1-1.2 | ASL | 0.91±0.13 (somatorsensory cortex) |
| Wegener et al, 2007 | 1.5 | ASL | ~1.19±0.13 (sensory and auditory cortex) |
| Mean±SEM |  |  | 1.29±0.05 |

**S2 Table. Physiological data (Mean±SEM; n = 12)** **obtained prior to hypercapnia and before KCl injection.** *significantly different from the value measured prior to hypercapnia; p<0.05.

|  | **pCO2 (mmHg)** | **pO2 (mmHg)** | **PH** | **MABP (mmHg)** | **Heart rate (beat/min)** |
| --- | --- | --- | --- | --- | --- |
| **Prior to hypercapnia** | 31.2±1.7 | 147.9±7.2 | 7.497±0.011 | 97.9±3.6 | 367.8±8.0 |
| **Prior to KCL injection** | 32.6±1.5 | 171.6.2±12.2 | 7.385±0.023* | 80.5±6.2* | 367.3±14.5 |

S3 Table. Summary of CC between rCBF and rR_1_^CBF^ as well as CC between rCBF and rR_1_^app^. Temporal correlation coefficient (CC) between rCBF and rR_1_^CBF^ measurements (CC_rR1CBF,rCBF_ ) as well as CC between the rCBF and rR_1_^app^ measurements (CC_rR1app,rCBF_ ) under hypercapnia and ischemia conditions based on the data analysis of region of interest located in the surface cortical region.

| **Ischemia** (n=5) **Hypercapnia** (n=3) |
| --- |
| **No. CC_rR1_^CBF^, _rCBF_ CC_rR1_^app^, _rCBF_ CC_rR1_^CBF^, _rCBF_ CC_rR1_^app^, _rCBF_** |
| 1 0.960 0.929 0.970 0.963  2 0.983 0.968 0.985 0.974  3 0.964 0.962 0.996 0.995  4 0.946 0.937  5 0.992 0.991 |
| **Mean 0.969±0.008 0.958±0.011 0.984 ±0.008 0.977±0.010**  **±SEM** |
| Paired t-test p = 0.10 p = 0.17 |

**Supporting Information: Figures**

**S1 Fig.** **Simulation results of B/A ratio in Eq. (5) in the main body text as a function of arterial transit time.** Simulation parameters: T_1_^app^ is 2.08 sec based on our study; T_1a_ = 2.38 sec taken from the literature [[2](#_ENREF_2)]; f is 1ml/g/min and λ is 0.9ml/g.

**S2 Fig. Temperature changes in rat brain and determine tissue-specific R_1_^temp^ dependence coefficient m’**. (a) and (b) Averaged brain temperature changes and standard error bars measured during (Stage 2) and after (Stages 3, 4 and 5) the induction of (a) 7-minute hypercapnia (n=3) and (b) one-minute ischemia (n=5) perturbation. (c) The inverse correlation between the temperature dependent R_1_ change (∆R_1_^temp^) and brain temperature change (∆T) measured after the KCl injection (i.e., CBF=0) from a representative rat. (d) Correlation between the averaged ∆R_1_^app^ measured by the SR-T_1_ MRI method versus (rCBF-1). The vertical bars indicate the standard error of the mean (SEM) (n=5).

S3 Fig. Time courses of rR_1_^app^ and rR_1_^CBF^ for hypercapnia and ischemia experiments in a representative rat. Time courses of relative R_1_^app^ (rR_1_^app^; solid lines) and R_1_^CBF^ (rR_1_^CBF^; dash lines) measured by the SR-T_1_ MRI method before, during and after (a) hypercapnia and (b) ischemia perturbation from a representative rat (data extracted from a region of interest). The black bar indicates the duration of (a) hypercapnia (7 minutes) and (b) ischemia (1 minute).

S4 Fig. Temporal responses comparison of rR_1_^app^, rR_1_^CBF^ and rCBF. Comparison of temporal responses of rR_1_^app^, rR_1_^CBF^ based on ROI analysis results and rCBF measured under (a) 7-minute hypercapnia (n=3), (b) the one-minute ischemia (n=5) perturbation condition, respectively. The first data point on the left side represents the control stage (Stage 1) and serves as a reference for normalization of other data points. The second data point stands for the averaged results measured under (a) hypercapnia or (b) ischemia perturbation (i.e., Stage 2). The third, fourth and fifth data points present the measurement results during three post-perturbation stages (Stages 3, 4 and 5). The vertical lines indicate the standard error of the mean (SEM).

**Supporting Information: References**

1. Herscovitch P, Raichle ME. What is the correct value for the brain--blood partition coefficient for water? J Cereb Blood Flow Metab. 1985;5(1):65-9. PubMed PMID: 3871783.

2. Dobre MC, Ugurbil K, Marjanska M. Determination of blood longitudinal relaxation time T_1_ at high magnetic field strengths. Magn Reson Imaging. 2007;25(5):733-5. PubMed PMID: 17540286.

3. Quesson B, de Zwart JA, Moonen CT. Magnetic resonance temperature imaging for guidance of thermotherapy. J Magn Reson Imaging. 2000;12(4):525-33. PubMed PMID: 11042633.

4. Parker DL, Smith V, Sheldon P, Crooks LE, Fussell L. Temperature distribution measurements in two-dimensional NMR imaging. Med Phys. 1983;10(3):321-5. PubMed PMID: 6877179.

5. Dickinson RJ, Hall AS, Hind AJ, Young IR. Measurement of changes in tissue temperature using MR imaging. J Comput Assist Tomogr. 1986;10(3):468-72. PubMed PMID: 3700752.

6. Botnar R. Interventional Magnetic Resonance Imaging, chapter 21 Temperature sensitive MR sequences. Berlin: Springer.: Springer Berlin Heidelberg; 1998.

7. Yablonskiy DA, Ackerman JJ, Raichle ME. Coupling between changes in human brain temperature and oxidative metabolism during prolonged visual stimulation. Proc Natl Acad Sci U S A. 2000;97(13):7603-8. PubMed PMID: 10861022.

8. Wang X, Zhu XH, Zhang Y, Chen W. Large enhancement of perfusion contribution on fMRI signal. J Cereb Blood Flow Metab. 2012;32(5):907-18. PubMed PMID: 22395206.

9. Duck FA. Physical Properties of Tissues: A Comprehensive Reference Book: Academic Press; 1990. 336 p.

10. Lewa CJ, Majewska Z. Temperature relationships of proton spin-lattice relaxation time T1 in biological tissues. Bulletin du cancer. 1980;67(5):525-30. PubMed PMID: 6260272.
